# Validating alcohol screening scores as measures of change

**DOI:** 10.1186/1940-0640-8-S1-A14

**Published:** 2013-09-04

**Authors:** Katharine A Bradley, Anna D Rubinsky, Emily C Williams, Gwen T Lapham, Laura J Chavez, Daniel R Kivlahan

**Affiliations:** 1 Group Health Research Institute, Seattle, WA, USA; 2 Health Services Research & Development (HSR&D) Northwest Center of Excellence, Veterans Affairs (VA) Puget Sound Health Care System, Seattle, WA, USA; 3 Center of Excellence in Substance Abuse Treatment and Education (CESATE), Veterans Affairs (VA) Puget Sound Health Care System, Seattle, WA, USA; 4 Department of Medicine, University of Washington, Seattle, WA, USA; 5 Department of Health Services, University of Washington, Seattle, WA, USA; 6 Center for Health Care Evaluation, Veterans Affairs (VA) Palo Alto Health Care System, Palo Alto, CA, USA; 7 Department of Psychiatry and Behavioral Sciences, University of Washington, Seattle, WA, USA

## Introduction

Evaluation of SBI implementation requires validated measures for monitoring drinking outcomes. The AUDIT-C is a validated alcohol screen, but the AUDIT-C’s validity as a measure of changes in drinking is unknown. This study evaluated whether changes in AUDIT-C scores at repeat annual screening have predictive validity in patients with and without documented BI (which could motivate under-reporting at follow-up).

## Methods

This retrospective cohort study used US Veterans Health Administration (VA) and Medicare data. VA outpatients (n 486,115) were eligible if they were screened with the AUDIT-C on 2 occasions (>12 months apart). Patients were categorized into 5 alcohol screening groups at each time: two groups with negative screens (nondrinkers and low risk drinkers) and 3 groups who screened positive for mild, moderate or severe misuse. Three outcomes were assessed in the year after patients’ 2^nd^ AUDIT-Cs: 1) HDL cholesterol, an alcohol biomarker; 2) trauma, and 3) hospitalization for liver disease, pancreatitis or upper GI bleeding. Regression analyses evaluated each outcome in groups of patients whose screening results changed or remained the same (25 groups) in patients with and without BI documented in the electronic medical record.

## Results

Changes in AUDIT-C scores were generally associated with expected changes in all 3 outcomes. For example, patients with severe alcohol misuse at baseline who resolved alcohol misuse had lower HDLs at follow-up (e.g. 44.8 mg/dl) than those with persistent severe alcohol misuse (55.0 mg/dl), and vice versa. One exception was that patients who resolved alcohol misuse did not have lower rates of trauma in the following year. Results were similar in those with and without documented BI.

## Conclusions

Changes in AUDIT-C scores have predictive validity, including after BI. This suggests AUDIT-C screens at follow-up could be used to evaluate and compare the effectiveness of different approaches to implementing alcohol interventions.

